# Editorial: Brain-inspired Hyperdimensional Computing: Algorithms, models, and architectures

**DOI:** 10.3389/fnins.2022.1102568

**Published:** 2022-12-08

**Authors:** Xun Jiao, Abbas Rahimi, Cornelia Fermüller, John Yiannis Aloimonos

**Affiliations:** ^1^Department of Electrical and Computer Engineering, Villanova University, Villanova, PA, United States; ^2^IBM Research - Zurich, Rüschlikon, Switzerland; ^3^Institute for Advance Computer Studies, University of Maryland, College Park, MD, United States; ^4^Department of Computer Science, University of Maryland, College Park, MD, United States

**Keywords:** Hyperdimensional (HD) Computing, hardware, brain-inspired computing, Artificial Intelligence, neuromorphic computing

The field of Hyperdimensional (HD) Computing, a.k.a. Vector Symbolic Architectures (VSA), is founded on the notion that the mind can be modeled by computing with high-dimensional vectors. Such vectors capture well the phenomena apparent in the ensemble activity of large populations of neurons in the brain. HD Computing has recently received increased interest from researchers in a variety of disciplines related to computer science and engineering. This is because of its promise as a framework for Artificial Intelligence (AI).

The power of HD Computing is due to statistical properties of high dimensional spaces, where two random vectors are likely to be nearly perpendicular. This makes it possible for multiple vectors representing different symbols (or concepts or images or sounds etc.) to be super-imposed into a single vector, in which the constituent components remain intact. This property is not shared by traditional computing with numbers. Three simple algebraic operations on vectors (addition, multiplication, and permutation) produce new vectors of the same dimensionality. These vectors in turn serve as components of subsequent operations or are stored in memory, which is the system's model of the world and is interpreted with the same three operations.

Neural networks form the backbone of today's AI. They achieve powerful implicit vector computations through self-organization using simple local operations within neuronal “units,” along with learning rules. However, the neural network approach suffers from several difficulties, such as lack of generalization to real-world situations outside the training set, high energy consumption and the need for large amounts of training data. By contrast, HD Computing provides a framework for computing in a distributed representation where the encodings and transformations are defined mathematically. Since they are not tied to local neuronal mechanisms, it becomes possible to understand encoding schemes and transformations independent from the neural network parametrization.

Addition is used to bundle elements together into a set, multiplication is used for variable binding or encoding spatial relations and computing transformations, and permutation is used for encoding order. Together these operations allow for combining information in rich and flexible ways that enable applications ranging from text processing and language identification to robotics and visual scene analysis. While the ideas of HD Computing go back 30 years, there has been a continuous string of accomplishments over the past 15 years. These have opened new horizons for research. The number of publications in this area is now growing exponentially.

Recent research has led to foundational theoretical advances, giving us a better understanding of the advantages of encoding in high-dimensions, the information capacity of high-dimensional vectors, and new algorithms for encoding and decoding. These have facilitated exciting applications in areas such as visual scene analysis, language identification, and robotics. HD Computing and VSAs are also an excellent fit for implementations on unconventional hardware, such as in-memory computing or neuromorphic hardware. Since they are computationally universal, they can act as a framework for computing with distributed representations and act as the abstraction layer for emerging computing hardware. HD arithmetic operations and search can easily be accelerated in embedded hardware leading to very low-power and high-performance implementations. Thus, another exciting active research area is about hardware solutions, as is reflected in two of the papers in this Research Topic (the third and fourth paper).

As HD Computing is searching for ways to formulate all technical problems in AI in a unifying framework, the field is advancing one step at a time, solving specific problems in various domains. It is in this spirit that we are happy to present this Research Topic on Hyper Dimensional Computing.

The first article of this Research Topic (Pale et al.) targets the challenging problem of real-time continuous epileptic seizure detection, where patients typically possess a high variability in their electroencephalogram (EEG) patterns. The paper proposes a novel semi-supervised learning approach based on a multi-centroid HDC model for detecting epileptic seizure, which leads to significantly improved performance when compared to a simple single-centroid HDC model. Further, the multi-centroid approach has shown promising performance for imbalance datasets, which is a typical situation for real-life datasets.

In HDC, the key-value superposition vector is a memory which can then be queried for the value of any of the keys, but the result of the query is approximate. The second article by Teeters et al. demonstrates that there is a better solution of an associative memory than using the superposition vector in a regime with a large number of key-value pairs. Associative memory (specifically versions of the Kanerva's Sparse Distributed Memory) maps the key vectors to value vectors while requiring lower memory to obtain the same reliability as the superposition vector.

The third article by Zou et al., proposes an end-to-end HD framework for processing signals recorded by event-based neuromorphic vision sensors. The gist lies in encoding the incoming asynchronous signal without binning it into images, while preserving the spatial and temporal data correlation. For training a robust scheme is proposed that implements a soft association to classes and allows incorporating unlabeled data. The method is implemented on FPGAs and demonstrated to outperform previous works in robustness for the tasks of classifying objects and associating 3D motion with event memories in a driving scenario.

Some of the operations required for HD computing, when mapped to parallel processing-in-memory architecture have been challenging to parallelize. The fourth article by Morris et al., explores stochastic computing to solve this bottleneck and improve the energy efficiency of HD Computing in deep learning tasks. The authors show that conventional multiplication can be replaced with a stochastic multiplier operation that leverages a set of parallel AND or XOR operations, which they use in HD encoding and similarity search operations. The approach is demonstrated on clustering and for several classification tasks using speech signals, images, and video.

With the hope that the community will find this Research Topic useful, we would like to extend our thanks to the all the contributors, the reviewers and the Frontiers dedicated staff.

## Author contributions

All authors listed have made a substantial, direct, and intellectual contribution to the work and approved it for publication.

